# Glucocorticoid receptor modulation as a host-directed strategy for inflammatory priming across chronic disease states

**DOI:** 10.3389/fphar.2026.1931270

**Published:** 2026-10-19

**Authors:** Richard A. Slayden, Ronald B. Tjalkens, Neil D. Theise, Christopher D. Verrico, Estefania P. Azevedo, John H. Van Drie, Michael T. Bardo, Mark A. Prendergast, Christopher M. Butt, Randice Altschul, Sujit S. Nair, Dimple Chakravarty, Ashutosh K. Tewari

**Affiliations:** 1 Department of Microbiology, Immunology and Pathology, Colorado State University, Fort Collins, CO, United States; 2 Neurodegenerative Disease Research Center, Barrow Neurological Institute, Phoenix, AZ, United States; 3 Department of Pathology, NYU Grossman School of Medicine, New York University, New York, NY, United States; 4 Baylor College of Medicine, Houston, TX, United States; 5 Department of Neuroscience, Medical University of South Carolina, Charleston, SC, United States; 6 Van Drie Research LLC, Grand Rapids, MI, United States; 7 Department of Psychology, University of Kentucky, Lexington, KY, United States; 8 28bio LLC, Durham, NC, United States; 9 Pop Test Oncology LLC, Cliffside Park, NJ, United States; 10 Department of Urology, Icahn School of Medicine at Mount Sinai, New York, NY, United States

**Keywords:** glucocorticoid receptor (GR) modulation, HPA axis dysregulation, inflammatory priming, neuroinflammation, NF-κB signaling

## Abstract

Chronic inflammation is increasingly recognized as a shared feature across neuropsychiatric, neurodegenerative, metabolic, oncologic, and post-infectious disorders, yet treatments mainly target downstream effects. Evidence suggests dysregulated glucocorticoid receptor (GR; encoded by *NR3C1*) signaling may regulate inflammation upstream, linking stress, HPA axis dysfunction, altered neuroimmune signaling, NF-κB activation, and ongoing inflammation. We review evidence supporting selective modulation of GR as a host-directed therapy targeting this shared mechanism. Across experimental systems, GR modulation is associated with attenuation of NF-κB-associated inflammatory signaling while preserving host-defense and repair-associated pathways. Exploratory clinical observations suggest that biological and therapeutic responses may depend on the degree of GR modulation, providing a hypothesis for prospective evaluation of a potential therapeutic window. Despite remaining questions, convergent evidence views dysregulated GR signaling as a promising upstream target across conditions like post-infectious inflammation, neuropsychiatric disorders, addiction, metabolic disease, and some cancers. We propose a biomarker-guided framework to evaluate selective GR modulation to restore the balance of inflammation in patient groups, rather than just suppressing downstream effects.

## Introduction

Therapeutic development across neuropsychiatric, neurodegenerative, metabolic, oncologic, and post-infectious diseases has historically proceeded within disease-specific frameworks focused on downstream manifestations such as neurotransmitter imbalance, protein aggregation, tumor proliferation, metabolic dysregulation, or persistent inflammatory sequelae. While this reductionist approach has generated important advances, it has also created limitations for host-directed therapeutics, which target common biological mechanisms operating across disease boundaries. Increasingly, evidence from neuroscience, immunology, endocrinology, and systems biology suggests that many diseases and chronic disorders share convergent pathways involving persistent inflammatory activation, stress-axis dysregulation, maladaptive neuroimmune signaling, and chronic inflammatory priming (Sorrells et al., 2009; Frank et al., 2013; Heneka et al., 2015; Miller and Raison, 2016; Guzman-Martinez et al., 2019).

Glucocorticoid receptor (GR; encoded by *NR3C1*) signaling is a key interface among environmental stressors, endocrine regulation, immune function, and tissue homeostasis (Sorrells et al., 2009; Frank et al., 2013). While traditionally viewed as anti-inflammatory due to the clinical use of corticosteroids, evidence shows that GR signaling effects depend on context (Sorrells et al., 2009; Frank et al., 2013). Acute activation suppresses inflammation and restores homeostasis, but chronic or dysregulated activation may sensitize inflammatory responses, modulate microglial activity, and amplify immune activation (Sorrells et al., 2009; Ros-Bernal et al., 2011). Glucocorticoids can serve as neuroendocrine signals, preparing the immune system for future challenges by acting as “alarm signals of danger” that prime innate immune pathways and lower thresholds for inflammation (Frank et al., 2015). Stress-induced DAMPs, such as HMGB1, also contribute to neuroinflammatory priming and heightened responses later (Frank et al., 2015). Instead of merely suppressing immunity, glucocorticoids participate in a dynamic system that influences inflammatory sensitivity and resilience and, under certain conditions, can exacerbate CNS inflammation, especially after stress, injury, or inflammatory stimuli (Sorrells et al., 2009).

This context dependence is central to the therapeutic hypothesis proposed here. GR signaling cannot be characterized as uniformly anti- or pro-inflammatory, as its effects depend on the magnitude and duration of glucocorticoid exposure, cellular context, receptor sensitivity, and the inflammatory state of the tissue. Acute physiological GR activation generally exerts anti-inflammatory effects, whereas prolonged or dysregulated glucocorticoid exposure can, under defined conditions, alter subsequent innate immune responsiveness and contribute to inflammatory priming, particularly within the CNS and neuroimmune systems. Rather, prolonged or dysregulated GR activation may alter receptor sensitivity and cellular inflammatory thresholds, contributing to a primed state in which subsequent challenges produce exaggerated responses. Partial pharmacological modulation of this dysregulated signaling state could therefore attenuate maladaptive inflammatory priming while preserving physiological GR functions required for immune regulation and homeostasis. In this framework, the therapeutic objective is restoration of regulatory balance rather than maximal GR antagonism. Although much of the mechanistic framework linking HPA-axis dysregulation, GR signaling, and inflammatory priming has been developed in the CNS and neuroimmune systems, emerging experimental evidence indicates that the consequences of GR modulation extend beyond the CNS. PT150 (formerly Org 34517) is a small-molecule glucocorticoid receptor (GR)-directed modulator originally developed as a GR antagonist for neuropsychiatric indications. Pharmacological studies indicate that PT150 interacts with GR and alters receptor-dependent transcriptional signaling, rather than completely suppressing physiological glucocorticoid activity. Because GR functionally interacts with inflammatory transcriptional networks, including NF-κB, modulating GR activity provides a plausible mechanism by which PT150 may influence downstream inflammatory signaling. However, these effects should not be interpreted as evidence that PT150 directly inhibits NF-κB, and the precise molecular mechanisms linking PT150–GR interaction to the inflammatory responses observed across experimental systems remain incompletely defined. Studies of PT150 in viral pneumonia, for example, support the potential relevance of GR-directed host modulation in peripheral inflammatory and infectious disease settings (Rocha et al., 2022).

These observations have important implications for understanding chronic disease. Many chronic disorders exhibit persistent inflammatory activation and altered HPA-axis function (Heneka et al., 2015; Koob and Volkow, 2016; Miller and Raison, 2016; van der Valk et al., 2018; Guzman-Martinez et al., 2019; Walsh et al., 2021; Rocha et al., 2022). The strength and nature of the supporting evidence nevertheless differ across disease settings, ranging from established mechanistic and translational observations in neuroimmune, neuropsychiatric, and infectious/inflammatory contexts to more preliminary evidence in several emerging indications. This supports the concept that inflammatory priming is a common biological process that affects mood, cognition, behavior, metabolism, and disease progression across diverse disorders (Miller and Raison, 2016).

Within this framework, dysregulated GR signaling may serve as an upstream regulatory mechanism that influences transitions between inflammatory states across multiple disease contexts. Chronic psychosocial stress, acute and persistent infections, metabolic overload, tumor-associated inflammation, neurodegeneration, and substance withdrawal all engage HPA-axis pathways that can alter GR-dependent signaling (Frank et al., 2013; Koob and Volkow, 2016; van der Valk et al., 2018). Experimental studies demonstrate that chronic stress can potentiate NF-κB activation and inflammatory responsiveness in the central nervous system, providing a mechanistic bridge between stress-axis dysregulation and chronic inflammatory amplification (Munhoz et al., 2006). Through interactions with NF-κB-associated transcriptional networks, inflammatory cytokine pathways, danger-associated molecular signaling, and microglial activation states, dysregulated GR signaling may contribute to the establishment of persistent inflammatory priming that amplifies downstream tissue injury and disease progression (Tsatsanis et al., 2006; Sorrells et al., 2009; Ros-Bernal et al., 2011; Frank et al., 2015; Picard et al., 2021). Importantly, this cross-disease framework represents a biological hypothesis regarding shared mechanisms of inflammatory priming and should not be interpreted as evidence that GR-directed pharmacological modulation has established therapeutic efficacy across each of the disease states discussed.

## Dysregulated GR signaling and inflammatory priming networks

The central hypothesis of this perspective is that dysregulated GR signaling contributes to the establishment and maintenance of chronic inflammatory priming across multiple disease contexts. Here, dysregulated GR signaling refers to a context-dependent departure from normal homeostatic GR regulation in which the magnitude, duration, sensitivity, or downstream consequences of GR signaling become maladaptive (Lockett et al., 2024). Inflammatory priming refers to a persistent or sensitized biological state in which prior stress, inflammatory, infectious, or other physiological exposures lower the threshold for, or increase the magnitude or duration of, inflammatory responses to subsequent challenges. Rather than a simple linear pathway, this process involves dynamic interactions among stress-responsive neuroendocrine systems, innate immune signaling networks, inflammatory transcriptional regulators, and tissue-specific cellular responses (Tsatsanis et al., 2006; Sorrells et al., 2009; Frank et al., 2013; Miller and Raison, 2016). Figure 1 summarizes the proposed framework in which diverse biological stressors converge on HPA-axis dysregulation, altered GR signaling, inflammatory priming, and downstream disease-specific manifestations.

**FIGURE 1:**
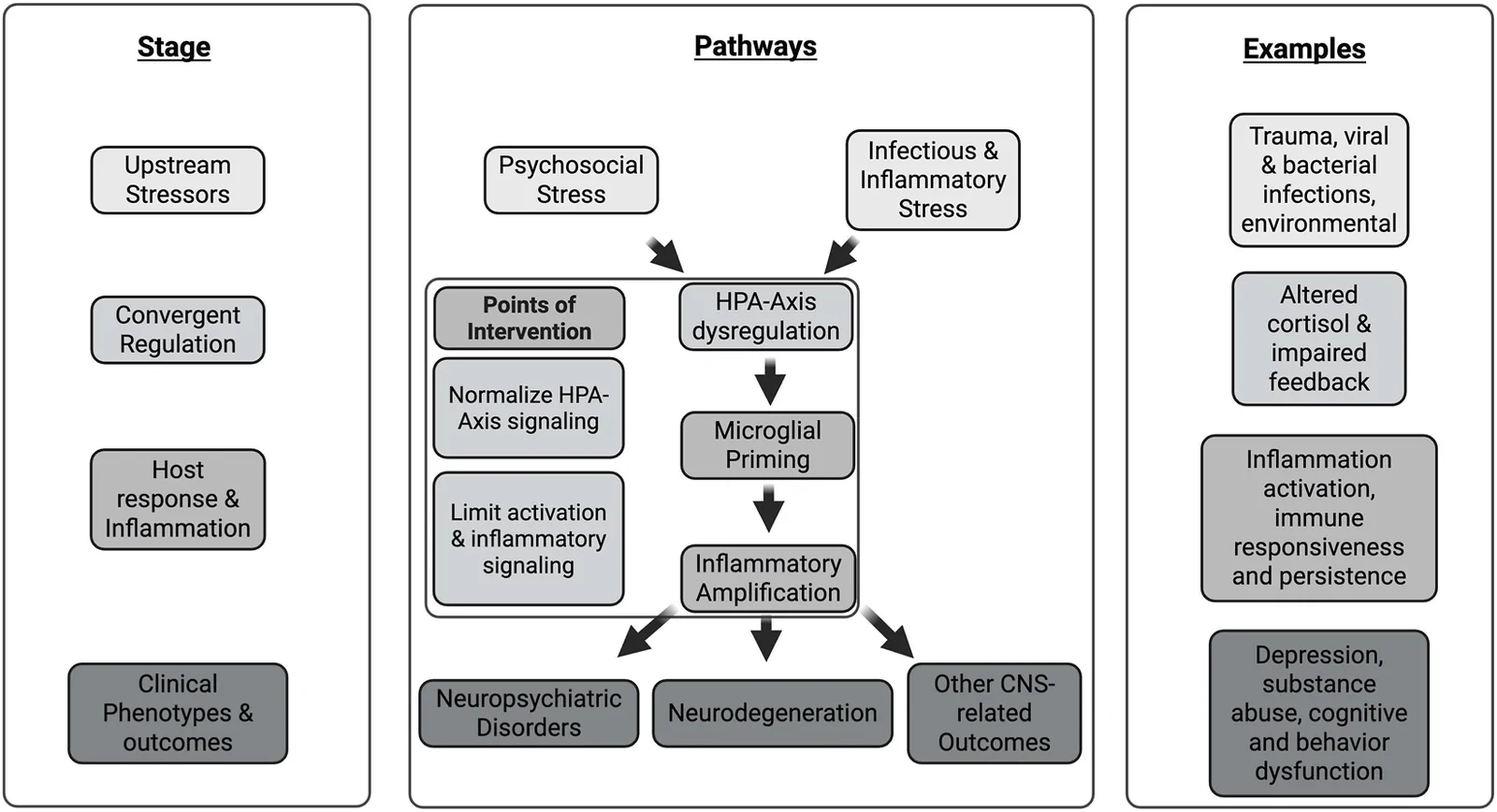
Conceptual framework linking stress-associated dysregulation to inflammatory priming and CNS-related outcomes. Psychosocial and infectious/inflammatory stressors may converge on hypothalamic-pituitary-adrenal (HPA) axis dysregulation and altered glucocorticoid signaling, contributing to microglial priming and amplification of inflammatory responses. Persistent or sensitized inflammatory signaling may, in turn, contribute to neuropsychiatric, neurodegenerative, and other CNS-related phenotypes. The framework also identifies potential points for therapeutic intervention, including modulation of HPA-axis signaling and attenuation of inflammatory priming and amplification. The relationships shown are conceptual and are not intended to imply a single linear mechanism or equivalent levels of evidence across the disease contexts represented.

Various biological stressors activate the hypothalamic-pituitary-adrenal (HPA) axis and modify glucocorticoid signaling, affecting inflammation and immune responses (Frank et al., 2013; Koob and Volkow, 2016; van der Valk et al., 2018). While acute glucocorticoid receptor (GR) activation aids in restoring balance, prolonged or dysregulated activation can influence inflammatory responses through immune priming, changes in receptor sensitivity, and transcriptional regulation (Sorrells et al., 2009; Frank et al., 2015). Microglia, crucial at the stress-inflammation interface, become sensitized after glucocorticoid exposure, leading to exaggerated inflammation (Sorrells et al., 2009; Ros-Bernal et al., 2011; Frank et al., 2013; Frank et al., 2015). Dysregulated GR signaling links stress pathways to persistent inflammation across diseases, explaining how triggers such as stress, infection, or substance withdrawal can cause prolonged inflammatory states.

At the molecular level, NF-κB functions as a major integrator of inflammatory signaling and is one of the most consistently implicated pathways downstream of stress-associated inflammatory priming (Tsatsanis et al., 2006; Sorrells et al., 2009). Chronic stress exposure has been experimentally shown to enhance NF-κB responsiveness in the frontal cortex and hippocampus, thereby increasing susceptibility to amplified inflammatory responses following subsequent immune challenge (Munhoz et al., 2006). Interactions among GR signaling, NF-κB activation, cytokine networks, and danger-associated molecular pattern signaling may therefore contribute to self-reinforcing inflammatory states that persist beyond the initiating stimulus (Sorrells et al., 2009; Frank et al., 2015).

Cyclooxygenase-2 (COX-2/PTGS2) represents one downstream component of this broader inflammatory network (Tsatsanis et al., 2006). Classical glucocorticoid signaling generally suppresses COX-2 expression as part of its anti-inflammatory activity (Brewer et al., 2003), although the relationship between GR signaling and inflammatory transcriptional programs is context dependent. Direct evidence that selective GR modulation by PT150 or PT157 acts through COX-2-dependent pathways remains incomplete. Accordingly, COX-2 is considered here as a potential downstream marker or integration node within the broader inflammatory response rather than as a direct target or obligatory mediator of GR-dependent therapeutic effects. Support for this network-based model is provided by unbiased transcriptomic and pathway enrichment analyses. Across independent REACTOME and MSigDB analyses, selective GR modulation with PT150 and PT157 consistently suppresses inflammatory pathways associated with NF-κB activation, TAK1/IKK signaling, p38 MAPK stress responses, IL-1, IL-6, TNF, and chemokine-associated signaling while preserving interferon-related pathways and enhancing selected repair-associated programs (Theise et al., 2020; Rocha et al., 2022).

## Conserved biological response to GR modulation across experimental systems

Selective GR modulation produces a remarkably consistent biological response across independent transcriptomic, protein-signaling, cellular, animal, translational, and early clinical studies (Figure 2). Although developed independently, these platforms consistently demonstrate attenuation of NF-κB-associated inflammatory pathways while preserving host-defense and tissue-repair programs (Theise et al., 2020; Morice et al., 2021; Rocha et al., 2022). This convergence supports dysregulated GR signaling as a tractable upstream regulator of inflammatory priming.

**FIGURE 2:**
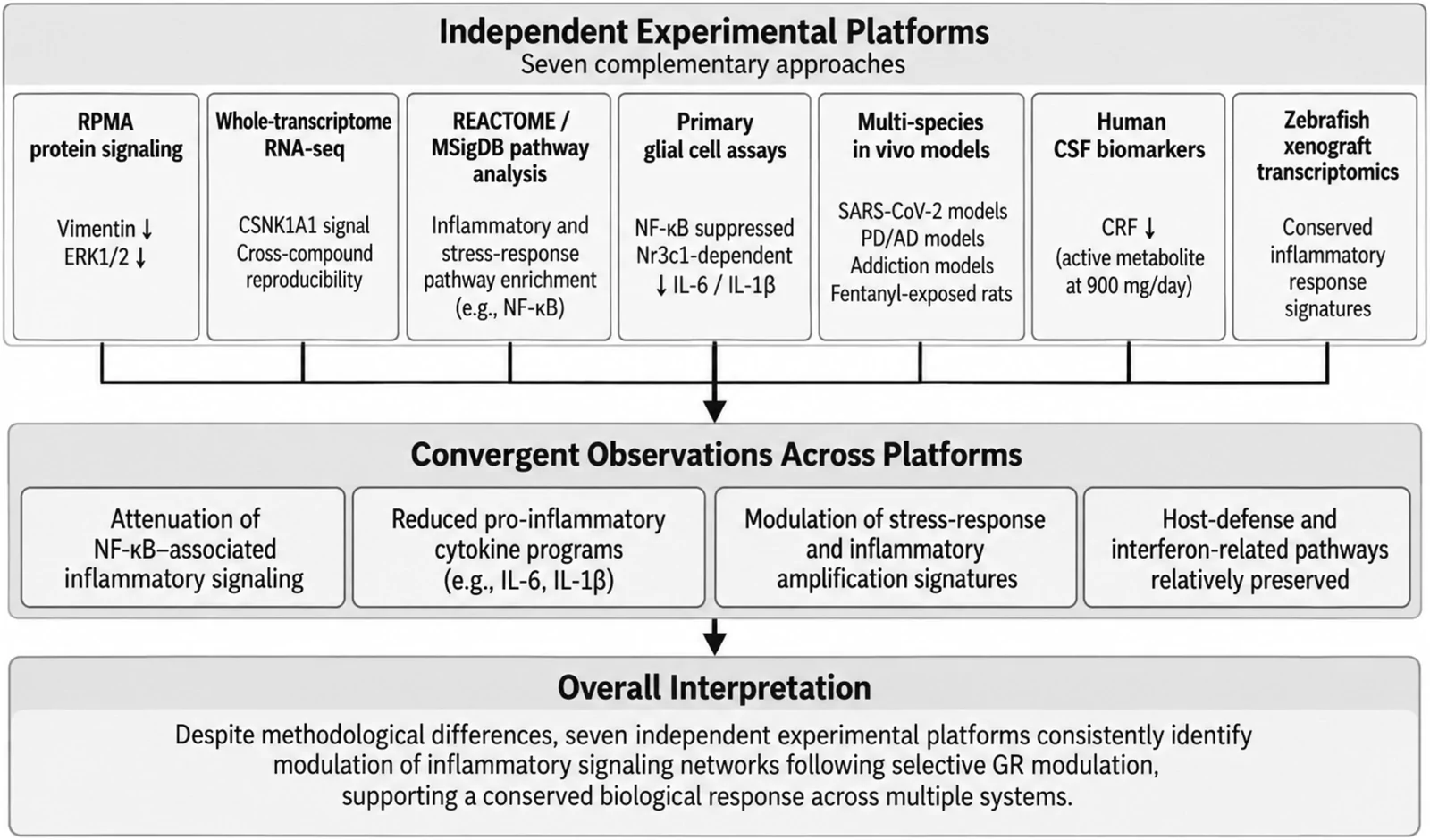
Convergent evidence for a conserved biological response to glucocorticoid receptor modulation across independent experimental platforms. Seven complementary experimental approaches—including reverse-phase protein microarray (RPMA) analysis, whole-transcriptome RNA sequencing, REACTOME/MSigDB pathway analysis, primary glial cell assays, multi-species *in vivo* models, human cerebrospinal fluid (CSF) biomarker studies, and zebrafish xenograft transcriptomics—provide independent evidence of biological responses associated with glucocorticoid receptor (GR) modulation. Despite differences in experimental system and methodology, convergent observations include attenuation of NF-κB-associated inflammatory signaling, reduced pro-inflammatory cytokine programs, modulation of stress-response and inflammatory amplification signatures, and relative preservation of host-defense and interferon-related pathways. Collectively, these findings support a conserved biological response to GR modulation across multiple experimental systems rather than validation of a single downstream molecular pathway or mechanism.

Preliminary evidence for GR dependence derives from non-peer-reviewed studies in primary glial cell systems reported in the PT150 Investigator’s Brochure. In these experiments, suppression of NF-κB activation and inflammatory cytokine expression following PT150 exposure was substantially diminished after knockdown of *Nr3c1* (PT150 Investigator’s Brochure, Edition 14, IND 133752). This observation is interpreted specifically as evidence of target dependence: reduction of GR abundance diminished the PT150-associated response, as would be expected if GR contributes to the pharmacological effect. The experiment does not imply that GR knockdown itself reproduces therapeutic modulation or establishes the physiological role of GR in inflammatory priming. Because these sponsor-associated studies have not undergone independent peer review, and potential knockdown-related or off-target effects have not been independently evaluated, these findings should be considered preliminary. Independent replication and mechanistic validation will be required to establish the specificity and generalizability of this effect.

Although these observations support involvement of GR signaling, they do not establish that the broader biological effects of PT150 or related compounds are exclusively GR-mediated. PT150 has also been characterized as a modulator of androgen receptor (AR) signaling (Theise et al., 2020), introducing an additional potential mechanism through which the compound may influence inflammatory responses. GR and AR are related steroid hormone receptors with overlapping transcriptional regulatory networks, and both may interact functionally with NF-κB-associated signaling. Consequently, some PT150-associated changes in inflammatory gene expression may reflect combined or context-dependent GR and AR modulation rather than GR activity alone. The relative contributions of GR, AR, and GR–AR crosstalk to these responses remain unresolved. In addition, GR signaling interacts with multiple transcriptional and signaling networks beyond NF-κB, including AP-1- and CREB-associated pathways, and can influence cellular responses through both genomic and non-genomic mechanisms. Accordingly, attenuation of NF-κB-associated inflammatory signaling is considered here as a reproducible downstream signature of the observed response rather than evidence of an exclusive linear GR-to-NF-κB mechanism.

Animal and translational studies further support the generalizability of this response. Across SARS-CoV-2 infection, addiction-related behaviors, neuroinflammatory models, and early human pharmacology studies, selective GR modulation consistently attenuates NF-κB-associated inflammatory signaling while preserving host-defense and tissue-repair pathways (Theise et al., 2020; Hammerslag et al., 2021; Rocha et al., 2022; Bardo et al., 2023; Radevski et al., 2023). These preclinical findings supported further clinical investigation of PT150 in viral or viral bacterial pneumonia, such as COVID-19, and an FDA IND was obtained for this indication, building on prior Phase I and Phase II human clinical experience with the compound in other indications. Although these systems differ substantially in biology and experimental design, they converge on a reproducible pattern of modulation of the inflammatory state rather than on generalized immunosuppression. Collectively, these observations support dysregulated GR signaling as a tractable upstream regulatory mechanism and provide a rationale for biomarker-guided clinical evaluation across disease contexts. Collectively, these orthogonal experimental platforms support a conserved biological response associated with pharmacological modulation of GR-centered signaling, characterized by attenuation of inflammatory amplification while preserving host-defense pathways. Although downstream manifestations vary across tissues and diseases, the reproducibility of this response identifies dysregulated GR signaling as a tractable upstream regulatory mechanism amenable to biomarker-guided investigation.

## Exploratory clinical observations suggest a potential therapeutic window for GR modulation

In a Phase II clinical study (Study 28104) evaluating two flexible-dose regimens of the GR-modulator, Org 34517 (PT150), in patients with major depressive disorder, the higher-dose regimen produced substantially greater elevations in circulating cortisol than the lower-dose regimen, consistent with greater compensatory HPA-axis activation following more extensive GR blockade (Organon Clinical Trial Report 28104). The lower-dose regimen produced substantially lower cortisol levels and showed more favorable outcomes on selected clinical measures and in exploratory analyses. Although assessment of the relationship between therapeutic response and baseline HPA-axis dysfunction was included among the study’s secondary objectives, the specific subgroup analyses were exploratory and were not prospectively powered to establish differential efficacy among biologically defined patient subgroups. These observations should therefore be regarded as hypothesis-generating rather than evidence of a predictive treatment biomarker. Nevertheless, they motivate prospective testing of whether biological measures of HPA-axis dysregulation can identify patient populations more likely to respond to selective GR modulation. This concept is consistent with precision-medicine approaches that seek to stratify clinically heterogeneous populations according to underlying biological state (Miller and Raison, 2016).

The clinical experience with PT150 further demonstrates that selective GR modulation is pharmacologically tractable and generally well tolerated in humans. Across clinical development programs (involving approximately 970 participants), no drug-related serious adverse events have been reported, including studies evaluating co-administration with ethanol and investigations involving repeated dosing (Morice et al., 2021; Vaughan et al., 2025) (PT150 Investigators Brochure Edition 14, IND 133752). These findings provide evidence that selective modulation of GR signaling can be achieved safely at clinically relevant exposures and support continued investigation of this therapeutic strategy. Importantly, the available human evidence primarily applies to PT150. Although PT157, a PT150 dimer, has demonstrated enhanced transcriptomic activity, favorable pharmacology, and encouraging preclinical tolerability, no human pharmacokinetic, safety, or efficacy data have yet to be reported (PT150 Investigators Brochure Edition 14). Accordingly, PT157 should be considered a next-generation preclinical candidate rather than a clinically validated therapeutic agent.

Current clinical evidence should be regarded as hypothesis-generating rather than confirmatory. Study 28104 was not designed to prospectively evaluate a therapeutic window or biomarker-guided treatment, but its observations provide a rationale for further investigation of partial GR modulation and biological-state–based patient stratification. Confirmation will require prospective dose-ranging studies incorporating endocrine and inflammatory biomarkers.

## Future directions

The evidence supporting GR modulation as a host-directed therapeutic strategy is derived from multiple independent experimental, translational, and clinical observations. Collectively, these studies support the biological plausibility of the proposed framework and suggest that dysregulated GR signaling may represent a tractable upstream regulator of inflammatory priming. Nevertheless, important limitations remain, and the current evidence should be interpreted as supporting a testable mechanistic hypothesis rather than establishing therapeutic efficacy across diverse disease contexts.

One limitation is that several mechanistic observations rely on internal datasets, exploratory analyses, or preclinical studies that have not yet undergone independent prospective validation. Where such non-peer-reviewed or sponsor-associated observations are discussed, they are considered hypothesis-supporting rather than independently validated evidence and should be interpreted accordingly.

Although unbiased transcriptomic analyses, REACTOME/MSigDB pathway enrichment studies, and orthogonal experimental platforms consistently identify suppression of NF-κB-associated inflammatory programs following selective GR modulation, additional validation by independent investigators will strengthen confidence in the generalizability of these findings (Theise et al., 2020; Rocha et al., 2022). Reproducibility across laboratories and disease models remains an important criterion for establishing whether these observations reflect a broadly conserved biological response.

Important disease-specific knowledge gaps also remain. Building on a strong CNS mechanistic foundation and complementary evidence from infectious and inflammatory disease models, post-infectious inflammatory syndromes, addiction-related disorders, and stress-associated neuropsychiatric conditions have relatively strong mechanistic and translational support. Evidence supporting applications in oncology, metabolic disease, amyotrophic lateral sclerosis (ALS), autism spectrum disorder (ASD), and other emerging indications remains substantially less mature. For example, ALS-associated observations are currently supported primarily by transcriptomic and computational analyses rather than validated *in vivo* efficacy studies. Similarly, hypotheses linking GR modulation to ASD involve interactions among neuroinflammation, stress biology, and folate-dependent neurodevelopmental pathways that have not yet been evaluated in prospective translational studies (Frye et al., 2013). These limitations do not invalidate the framework but define the current boundaries of evidence.

The available clinical observations provide preliminary insight into the biological effects of GR modulation in humans and are consistent with a potential therapeutic window characterized by partial rather than maximal receptor antagonism. However, the clinical study was not designed to test this hypothesis prospectively, and the subgroup analyses suggesting enhanced responses in individuals with stress-axis dysregulation remain exploratory (Organon Clinical Trial Report 28104). Replication in prospective trials specifically designed to evaluate biomarker-defined populations will be necessary before definitive conclusions can be drawn regarding optimal target engagement or clinical efficacy.

A central prediction of the proposed framework is that therapeutic responsiveness will depend more strongly on biological state than on disease classification. This prediction suggests that future studies should prioritize identifying and validating biomarkers that quantify GR-dependent inflammatory priming. Candidate biomarkers include cortisol awakening responses, dexamethasone suppression testing, adrenocorticotropic hormone (ACTH) concentrations, cerebrospinal fluid corticotropin-releasing factor (CRF), inflammatory cytokine profiles, NF-κB-associated transcriptional signatures, and broader transcriptomic measures of the inflammatory state (Frank et al., 2013; Miller and Raison, 2016). Validation of such biomarkers would facilitate prospective patient stratification and provide a mechanistic basis for evaluating treatment response.

Future clinical development should therefore focus on biomarker-guided trial designs incorporating longitudinal endocrine measurements, inflammatory profiling, dose-ranging strategies, and predefined enrichment criteria based on stress-axis dysregulation. Such studies would directly test the hypothesis that partial modulation of dysregulated GR signaling can restore inflammatory and neuroendocrine homeostasis more effectively than maximal receptor antagonism. Importantly, this approach aligns with broader trends in precision medicine, which increasingly seek to classify patients according to biological state rather than conventional diagnostic categories (Miller and Raison, 2016).

Ultimately, the central question is not whether GR modulation can be applied across multiple disease indications, but whether modulation of a shared upstream regulatory mechanism can reproducibly alter the inflammatory state in biomarker-defined patient populations. The answer to this question will determine whether GR modulation represents a broadly applicable host-directed therapeutic paradigm or a more context-dependent intervention. The existing evidence provides a compelling rationale for testing this hypothesis prospectively, but definitive validation remains an important objective for future investigation.

## Conclusion

The evidence reviewed in this Perspective is consistent with a unifying hypothesis: dysregulated glucocorticoid receptor (GR) signaling may function as an upstream regulator of inflammatory priming across diverse chronic disease states. Although neuropsychiatric disorders, neurodegenerative diseases, post-infectious syndromes, addiction, metabolic dysfunction, and selected malignancies differ substantially in clinical presentation, many share common features involving stress-axis dysregulation, altered neuroimmune signaling, and persistent inflammatory activation. From this perspective, selective GR modulation is not proposed as a strategy to treat multiple unrelated diseases with a single agent, but rather as an approach to influence a shared biological process that contributes to disease susceptibility, persistence, and progression.

A notable feature of the available evidence is the convergence of observations across independent experimental systems. Transcriptomic analyses, pathway-enrichment studies, primary glial cell experiments, animal models, translational biomarker investigations, and early human studies consistently identify modulation of NF-κB-associated inflammatory signaling while preserving host-defense and repair-associated pathways. Although the downstream manifestations vary across tissues and disease contexts, this reproducible pattern supports dysregulated GR signaling as a potentially tractable upstream regulator of inflammatory priming. Important uncertainties remain, including validation of specific downstream mediators, prospective confirmation of the proposed therapeutic window, and identification of biomarkers that reliably define GR-dependent inflammatory states. Accordingly, future clinical development should prioritize biomarker-guided trial designs that quantify inflammatory priming, stress-axis dysregulation, and GR-dependent signaling activity. Ultimately, the significance of this framework lies not in any individual compound or disease indication, but in the hypothesis that dysregulated GR signaling represents a shared upstream mechanism linking stress biology to chronic inflammatory disease. Rigorous prospective validation will determine whether modulation of this pathway can reproducibly restore inflammatory homeostasis in biologically defined patient populations.

## Data Availability

The raw data supporting the conclusions of this article will be made available by the authors, without undue reservation.

## References

[B1] Bardo M. T. Chandler C. M. Denehy E. D. Carper B. A. Prendergast M. A. Nolen T. L. (2023). Effect of the glucocorticoid receptor antagonist PT150 on acquisition and escalation of fentanyl self-administration following early-life stress. Exp. Clin. Psychopharmacol. 31, 362–369. 10.1037/pha0000577 35587421 PMC10084834

[B2] Brewer J. A. Khor B. Vogt S. K. Muglia L. M. Fujiwara H. Haegele K. E. (2003). T-cell glucocorticoid receptor is required to suppress COX-2-mediated lethal immune activation. Nat. Med. 9, 1318–1322. 10.1038/nm895 12949501

[B3] Frank M. G. Watkins L. R. Maier S. F. (2013). Stress-induced glucocorticoids as a neuroendocrine alarm signal of danger. Brain Behav. Immun. 33, 1–6. 10.1016/j.bbi.2013.02.004 23459026 PMC5652304

[B4] Frank M. G. Weber M. D. Watkins L. R. Maier S. F. (2015). Stress sounds the alarmin: the role of the danger-associated molecular pattern HMGB1 in stress-induced neuroinflammatory priming. Brain Behav. Immun. 48, 1–7. 10.1016/j.bbi.2015.03.010 25816800 PMC4508196

[B5] Frye R. E. Sequeira J. M. Quadros E. V. James S. J. Rossignol D. A. (2013). Cerebral folate receptor autoantibodies in autism spectrum disorder. Mol. Psychiatry 18, 369–381. 10.1038/mp.2011.175 22230883 PMC3578948

[B6] Guzman-Martinez L. Maccioni R. B. Andrade V. Navarrete L. P. Pastor M. G. Ramos-Escobar N. (2019). Neuroinflammation as a common feature of neurodegenerative disorders. Front. Pharmacol. 10, 1008. 10.3389/fphar.2019.01008 31572186 PMC6751310

[B7] Hammerslag L. R. Denehy E. D. Carper B. Nolen T. L. Prendergast M. A. Bardo M. T. (2021). Effects of the glucocorticoid receptor antagonist PT150 on stress-induced fentanyl seeking in male and female rats. Psychopharmacol. Berl. 238, 2439–2447. 10.1007/s00213-021-05865-0 34008048 PMC10323366

[B8] Heneka M. T. Carson M. J. El Khoury J. Landreth G. E. Brosseron F. Feinstein D. L. (2015). Neuroinflammation in Alzheimer's disease. Lancet Neurol. 14, 388–405. 10.1016/S1474-4422(15)70016-5 25792098 PMC5909703

[B9] Koob G. F. Volkow N. D. (2016). Neurobiology of addiction: a neurocircuitry analysis. Lancet Psychiatry 3, 760–773. 10.1016/S2215-0366(16)00104-8 27475769 PMC6135092

[B10] Lockett J. Inder W. J. Clifton V. L. (2024). The glucocorticoid receptor: isoforms, functions, and contribution to glucocorticoid sensitivity. Endocr. Rev. 45, 593–624. 10.1210/endrev/bnae008 38551091 PMC11244253

[B11] Miller A. H. Raison C. L. (2016). The role of inflammation in depression: from evolutionary imperative to modern treatment target. Nat. Rev. Immunol. 16, 22–34. 10.1038/nri.2015.5 26711676 PMC5542678

[B12] Morice C. Baker D. G. Patel M. M. Nolen T. L. Nowak K. Hirsch S. (2021). A randomized trial of safety and pharmacodynamic interactions between a selective glucocorticoid receptor antagonist, PT150, and ethanol in healthy volunteers. Sci. Rep. 11, 9876. 10.1038/s41598-021-88609-6 33972573 PMC8111026

[B13] Munhoz C. D. Lepsch L. B. Kawamoto E. M. Malta M. B. Lima Lde S. Avellar M. C. (2006). Chronic unpredictable stress exacerbates lipopolysaccharide-induced activation of nuclear factor-kappaB in the frontal cortex and hippocampus via glucocorticoid secretion. J. Neurosci. 26, 3813–3820. 10.1523/JNEUROSCI.4398-05.2006 16597735 PMC6674142

[B14] Picard K. Bisht K. Poggini S. Garofalo S. Golia M. T. Basilico B. (2021). Microglial-glucocorticoid receptor depletion alters the response of hippocampal microglia and neurons in a chronic unpredictable mild stress paradigm in female mice. Brain Behav. Immun. 97, 423–439. 10.1016/j.bbi.2021.07.022 34343616

[B15] Radevski M. E. Prendergast M. A. Bardo M. T. Akins C. K. (2023). PT150 blocks the rewarding properties of ethanol and attenuates ethanol-induced reduction of egg laying in coturnix quail. Psychopharmacol. Berl. 240, 295–301. 10.1007/s00213-022-06299-y 36607385

[B16] Rocha S. M. Fagre A. C. Latham A. S. Cummings J. E. Aboellail T. A. Reigan P. (2022). A novel glucocorticoid and androgen receptor modulator reduces viral entry and innate immune inflammatory responses in the Syrian hamster model of SARS-CoV-2 infection. Front. Immunol. 13, 811430. 10.3389/fimmu.2022.811430 35250984 PMC8889105

[B17] Ros-Bernal F. Hunot S. Herrero M. T. Parnadeau S. Corvol J. C. Lu L. (2011). Microglial glucocorticoid receptors play a pivotal role in regulating dopaminergic neurodegeneration in parkinsonism. Proc. Natl. Acad. Sci. U. S. A. 108, 6632–6637. 10.1073/pnas.1017820108 21467220 PMC3080980

[B18] Sorrells S. F. Caso J. R. Munhoz C. D. Sapolsky R. M. (2009). The stressed CNS: when glucocorticoids aggravate inflammation. Neuron 64, 33–39. 10.1016/j.neuron.2009.09.032 19840546 PMC4782919

[B19] Theise N. D. Arment A. R. Chakravarty D. Gregg J. M. H. Jacobson I. M. Jung K. H. (2020). Clinical stage molecule PT150 is a modulator of glucocorticoid and androgen receptors with antiviral activity against SARS-CoV-2. Cell. Cycle 19, 3632–3638. 10.1080/15384101.2020.1859752 33305659 PMC7738205

[B20] Tsatsanis C. Androulidaki A. Venihaki M. Margioris A. N. (2006). Signalling networks regulating cyclooxygenase-2. Int. J. Biochem. Cell. Biol. 38, 1654–1661. 10.1016/j.biocel.2006.03.021 16713323

[B21] Van Der Valk E. S. Savas M. Van Rossum E. F. C. (2018). Stress and obesity: are there more susceptible individuals? Curr. Obes. Rep. 7, 193–203. 10.1007/s13679-018-0306-y 29663153 PMC5958156

[B22] Vaughan A. Mensah C. K. Pennycuff J. Venneman S. S. Risborough V. Baker D. G. (2025). Pharmacokinetic interactions between ethanol and PT150, a selective glucocorticoid receptor antagonist, in healthy volunteers. J. Addict. Med. 20 (4), 409–415. 10.1097/ADM.0000000000001557 40794488

[B23] Walsh C. P. Bovbjerg D. H. Marsland A. L. (2021). Glucocorticoid resistance and beta2-adrenergic receptor signaling pathways promote peripheral pro-inflammatory conditions associated with chronic psychological stress: a systematic review across species. Neurosci. Biobehav Rev. 128, 117–135. 10.1016/j.neubiorev.2021.06.013 34116126 PMC8556675

